# Pregnancy outcomes among women with inflammatory bowel disease: A UK tertiary centre experience

**DOI:** 10.1007/s12664-024-01657-4

**Published:** 2024-09-02

**Authors:** Ruth Tunney, Eleanor Liu, Jimmy K. Limdi

**Affiliations:** 1 Department of Gastroenterology, Northern Care Alliance NHS Trust, Manchester, UK; 2 University of Manchester, Manchester, UK

**Keywords:** Inflammatory bowel disease, Pregnancy outcomes

## Abstract

**Background:**

Optimal management of inflammatory bowel disease (IBD) in pregnancy is associated with better pregnancy outcomes. We describe management of IBD during pregnancy and maternal and fetal outcomes of patients from a tertiary UK IBD centre.

**Methods:**

This is a retrospective observational cohort study of all pregnancies occurring between 2015 and 2021 in a large tertiary IBD centre in the UK. IBD activity and management prior to, during and after pregnancy were recorded along with pregnancy and neonatal outcomes. Associations between IBD-focused interventions and any adverse pregnancy outcomes, as well as the association between IBD severity and treatments and adverse maternofetal outcomes were assessed.

**Results:**

Pregnancies in 130 women with IBD were included for analysis. The mean maternal age at delivery was 30.5 (± 4.7) years. At conception, 73 women (56.2%) were in clinical remission and 24 (18.4%) were treated with a biologic agent. Active disease during pregnancy, measured by physician global assessment, was less frequent in women who were in clinical remission at conception, compared to those not in remission at conception (16/73 21.9% vs. 39/49 79.6%; data insufficient for eight women). Active IBD at conception was associated with pre-term birth (*p* = 0.04). Maternal corticosteroid use in any trimester was associated with low birth weight (T1 *p* = 0.02; T2 *p* = 0.005; T3 *p* = 0.007). Active disease (*p* = 0.008) and steroid use in the third trimester (*p* = 0.05) were both associated with neonatal infections up to six months after birth.

**Conclusion:**

Women in clinical remission at the time of conception have favorable outcomes, consistent with prospective observational studies. Our observations emphasize the importance of high quality IBD care for women pre and post-partum in line with international recommendations.

**Graphical Abstract:**

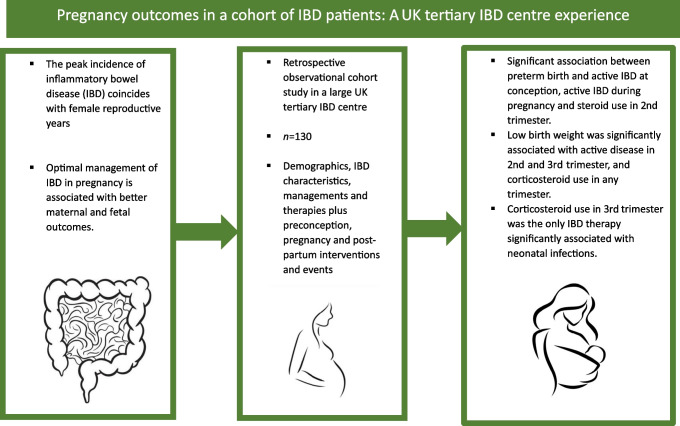

**Supplementary Information:**

The online version contains supplementary material available at 10.1007/s12664-024-01657-4.

## Introduction

Inflammatory bowel diseases (IBD), comprising Crohn’s disease (CD) and ulcerative colitis (UC) are chronic relapsing–remitting immune mediated inflammatory conditions affecting the gastrointestinal tract [1]. The peak incidence of IBD is between the second and fourth decades of life, coinciding with prime female reproductive years [1, 2]. IBD activity at the time of conception is a key determinant of the course of IBD during pregnancy and active disease is associated with adverse pregnancy-related outcomes such as miscarriage, intra-uterine growth restriction and pre-term birth [3]. Optimal disease control improves pregnancy related outcomes [3], comparable to the general, non-IBD population [4]. Multidisciplinary and holistic management of pregnant women with IBD is recommended, with specific attention to be paid to achieving clinical remission prior to conception, pre-conception counselling, joint obstetric-and IBD management and individualized decision-making regarding IBD medications during pregnancy and the post-partum period, [5–7]. Previous work has found significant variation in managing IBD in pregnancy [8]. In recent years, there has been an evolution in our therapeutic armamentarium and data from prospective observational studies provides reassurance and guidance on modern management of IBD in pregnancy [5, 6, 9-11]. The real-world impact of this data is less well-described. We aimed at describing pregnancy outcomes of women from a large tertiary IBD centre in the UK and comparing the standard of care in this centre to current guidelines [6].

## Methods

### Study design

This was a single-centre, retrospective observational uncontrolled cohort study, including consecutive IBD pregnancies occurring between January 1, 2015, and December 31, 2021, at the Northern Care Alliance NHS Trust, Manchester, a large tertiary IBD centre in the United Kingdom (UK) overseeing the care of 5000 people with IBD. Pregnant women of any gestational age and a confirmed diagnosis of IBD (CD, UC or IBD-unspecified [IBD-U]), undergoing follow-up at our institution were eligible for inclusion. Patients were excluded if they had a miscarriage before their initial obstetric booking appointment, insufficient data or incomplete information on pregnancy outcome. Pregnancies ending after December 31, 2021, were also excluded, representing our cut-off for data acquisition.

Data was collected from local IBD-pregnancy databases as well as gastroenterology and obstetric medical records. Vaginal births and cesarean sections (CS) were all included. Baseline demographic data, IBD-related data, including diagnosis, disease duration and phenotype (Montreal classification), previous IBD-related surgical history and stoma status was collected. Information on IBD management, physician global assessment (PGA) of disease activity and objective markers of disease activity (serum C-reactive protein [CRP] and fecal calprotectin [FC]), where available, were recorded in the six months prior to conception and in each trimester. Data on IBD-specific therapy (including aminosalicylates [5-ASA], corticosteroids, thiopurines and biologics) was collected at each trimester, including point of cessation of biologics during pregnancy where this occurred. Obstetric considerations, in particular, data on third trimester (T3) growth scans, venous thromboembolism (VTE) prophylaxis and delivery mode were recorded. Assessment for VTE prophylaxis was carried out by the antenatal obstetric team in accordance with national guidelines set by the Royal College of Obstetricians and Gynecologists, as recommended by the British Society of Gastroenterology (BSG) [6]. Pregnancy and neonatal adverse outcomes and maternal post-natal IBD outcomes, including IBD complications within 30 days of delivery, re-initiation of biologic therapy and IBD follow-up, were also recorded. This study was exempt from ethical approval, being a pragmatic audit of routinely collected clinical data.

### Outcomes

Our primary aim was to describe adverse pregnancy and neonatal outcomes, namely pre-term birth, low birth weight (LBW), congenital anomalies and neonatal infections within six months of life. Our secondary aims were to determine any associations between maternal IBD disease activity and in utero exposure to IBD medications with adverse pregnancy outcomes and to assess the provision of antenatal care for patients with IBD in line with UK guidance, endorsed by the BSG [6].

### Definitions

The first trimester (T1) was defined from the date of the last menstrual period until the 12th week of gestation, second trimester (T2) from the 13th to 28th week and T3 from 29th gestational week until delivery. Pre-term birth was defined as delivery at < 37 weeks' gestation. LBW was defined as a birthweight < 2500 g and small for gestational age (SGA) was defined as a birth weight of < 10th percentile for gestational age. Miscarriage was defined as fetal loss up to 23 + 6 gestation; any loss beyond this gestation was defined as stillbirth. Neonatal infections were defined as serious if the infant required hospitalization. IBD disease activity was assessed clinically by the investigators and defined using Physican’s Global Assessment as remission, mild, moderate or severe, based on review of the medical documentation, considering patient reported symptoms, bio-chemical markers of disease activity, IBD therapy and escalation and IBD-related hospitalization. IBD complications were defined as a flare of IBD symptoms requiring medical input and/or therapy change or escalation.

### Statistical Analysis

Quantitative variables were expressed as mean and ± SD. Qualitative variables were expressed as frequency and percentages. Independent *t*-test was used to compare the means for continuous data and Chi-square test and Fisher’s exact test used for comparing proportions of categorical data. Chi-square test was applied to analyze association between adverse pregnancy-related outcomes and neonatal outcomes, including preterm birth, emergency CS, LBW and congenital anomalies with either disease activity or IBD therapy use during pregnancy, steroid usage, pre-term birth and LBW. A *p* value < 0.05 was considered statistically significant.

## Results

### Patients and Baseline Characteristics

Data was collected for 130 pregnancies; (UC: 75/130 [57.7%], CD: 54/130 [41.5%] and IBD-U in one patient). Baseline demographics and IBD characteristics are set out in Table 1. All pregnancies were singleton except for one; this pregnancy resulted in the live birth of one neonate after fetal loss of the twin at eight weeks’ gestation. Mean duration of IBD diagnosis at the time of delivery was 7.1 ± 5.5 years (CD 9.4 ± 5.9; UC 5.3 ± 4.3; *p* =  < 0.01). Nine women (6.9%) had a previous ileocecal resection and three (2.3%) had a previous hemicolectomy for Crohn’s disease. Four patients had a previous sub-total colectomy (3.1%; 3/54 [5.6%] for CD and 1/75 [1.3%] for UC). There were no patients with ileal pouch-anal anastomosis (IPAA). Five patients (3.8%) had an ileostomy. Disease phenotype in UC was available for 74/75 patients; proctitis/E1 (22 [29.7%]), left-sided/E2 (25 [33.8%]) and extensive/E3 disease (27 [36.5%]). With Crohn’s disease, 16 (29.5%) had isolated colonic disease and 29 (53.7%) had ileo-colonic disease. Most Crohn’s patients (37 [68.5%]) had non-stricturing, non-penetrating disease. Seven women (13%) had perianal CD (Table 1).

**Table 1:** Baseline demographics and inflammatory bowel disease characteristics

Demographic/Characteristic	All IBD *n* = 130	UC *n* = 75 (57.7%)*	CD *n* = 54 (41.5%)	*p-*value
Maternal age at delivery, years, mean ± SD	30.5 ± 4.7	30.5 ± 4.5	30.6 ± 5.1	0.87
BMI	26.1 ± 5.8	25.2 ± 4.9	27.4 ± 6.8	0.01
Smoking status, *n* (%) Current Ex-smoker Never	14 (10.8) 19 (14.6) 97 (74.6)	3 (4) 7 (9.3) 65 (86.7)	11 (20.4) 12 (22.2) 31 (57.4)	< 0.01
Duration of IBD diagnosis, years, mean ± SD	7.1 ± 5.5	5.3 ± 4.3	9.4 ± 5.9	< 0.01
Previous IBD surgery, *n* (%)	16 (12.3)	1 (1.3)	15 (27.8)	
Stoma, *n *(%)	5 (3.8)	1 (1.3)	4 (7.4)	
Montreal E, *n* (%), Proctitis (E1)/Left-sided (E2)/Extensive (E3), *n *(%)		22 (29.7)/25 (33.8)/27 (36.5)		
Montreal L, *n* (%), Ileal (L1)/Colonic (L2)/Ileo-colonic (L3)/Upper GI (L4)			9 (16.7)/16 (29.6)/29 (53.7)/0 (0)	
Montreal B, *n* (%), Non-stricturing, non-penetrating (B1)/Stricturing (B2), Penetrating (B3)			37 (68.5)/11 (20.4)/6 (11.1)	
Perianal (p)			7 (13)	

^*^ Data on disease extent according to Montreal classification available for 74/75 patients

There was no statistical association between BMI, age at delivery, smoking status and the outcomes of congenital anomalies (*p* = 0.5018, *p* = 0.0819, *p* = 0.9341), LBW (*p* = 0.4494, *p* = 0.3609, *p* = 0.8633), SGA (*p* = 0.2365, *p* = 0.3692, *p* = 0.6647), neonatal infections (*p* = 0.1455, *p* = 0.3910, *p* = 0.9583), emergency CS (*p* = 0.4991, *p* = 0.7949, *p* = 0.4631) and preterm birth (*p* = 0.5345, *p* = 0.5573, *p* = 0.9316).

The mean BMI at conception was 26.1 ± 5.8 kg/m^2^ and mean maternal age at delivery was 30.5 ± 4.7 years; comparable for both CD and UC (*p* = 0.01 and *p* = 0.87 respectively). Active smoking was more frequent with CD than UC (11/54 [20.4%] vs. 3/75 [4%]) and a past history of smoking was also more frequent with CD (12/54 [22.2%] vs. UC 7/75 [9.3%], *p* =  < 0.01). Forty-nine women (38%) were nulliparous, 45 (34.9%) were primiparous and 36 (27.9%) were multiparous.

### Pregnancy-related Management

Active follow-up with an IBD specialist was in place at conception in 120/130 (92.3%) and 91/120 patients had been reviewed within six months prior to conception. Prior to conception, a documented discussion had taken place about family planning and optimal disease control for conception in 24/120 patients (20%). All 130 patients were actively under follow-up by IBD team during their pregnancy. During pregnancy, all women were under routine midwife review, in accordance with standard UK antenatal care. Additionally, 92/130 (70.8%) were also under obstetric review. The mean number of reviews by the IBD team during pregnancy was 2.3 (± 1.7). There was documented communication between the obstetric and IBD team in 59/130 cases (45.4%) although the record of clinical assessment is electronically available to clinicians caring for patients across specialities. Advice regarding the importance of inducing and maintaining remission and advice regarding IBD medications in pregnancy was documented in 75/130 cases (57.7%).

### IBD Disease Activity

IBD activity, stratified by diagnosis and trimester, is summarized in Table 2. Overall, 73/130 (56.2%; CD 34/54, [63%]; UC 39/75, [52%]) were in clinical remission at the time of conception and 49/130 had mild-moderate disease activity at conception, based on physician global assessment (37.8%; CD 19/54 [35.2%]; UC 29/75 [38.7%], IBD-U 1/1). There were no patients with severe disease activity at the time of conception and data was not available for the pre-conception period in 8/130 women. Of patients in clinical remission at the time of conception, 15/73 developed mild-moderate disease activity during pregnancy and one patient, with UC, developed severe disease activity.

**Table 2:** Inflammatory bowel disease activity at conception and during pregnancy*

Characteristic	Overall, *n* = 130*^1^	CD, *n* = 54^*1*^	UC, *n* = 75^*1*^	*p*-value^2^
**PGA disease activity—conception**				0.7
Remission	73 (60%)	34 (64%)	39 (57%)	
Mild	37 (30%)	15 (28%)	21 (31%)	
Moderate	12 (9.9%)	4 (7.5%)	8 (12%)	
**PGA disease activity T1**				0.003
Remission	81 (68%)	42 (81%)	39 (58%)	
Mild	27 (23%)	5 (9.6%)	22 (33%)	
Moderate	9 (7.6%)	3 (5.8%)	6 (9.0%)	
Severe	2 (1.7%)	2 (3.8%)	0 (0%)	
**PGA disease activity T2**				0.2
Remission	73 (66%)	37 (76%)	36 (59%)	
Mild	25 (23%)	8 (16%)	17 (28%)	
Moderate	10 (9.1%)	4 (8.2%)	6 (9.8%)	
Severe	2 (1.8%)	0 (0%)	2 (3.3%)	
**PGA disease activity T3**				0.4
Remission	75 (64%)	36 (71%)	39 (58%)	
Mild	24 (20%)	10 (20%)	14 (21%)	
Moderate	17 (14%)	5 (9.8%)	12 (18%)	
Severe	2 (1.7%)	0 (0%)	2 (3.0%)	

^*1*^*n* (%)

^*2*^Pearson's Chi-squared test; Fisher's exact test

*One patient in cohort had IBD-U diagnosis and was not included in the table, but included in the % calculations

Overall, 49/130 (37.7%) patients had mild-moderate disease at the time of conception. There was higher disease activity during pregnancy in this group, with 35/45 (77.8%) continuing to experience mild-moderate disease (CD = 10, UC = 25) and 4/45 (8.9%) with severe activity (CD = 2, UC = 2). Six women (4.6%, UC = 5, CD = 1) had an IBD-related hospital admission during pregnancy. Four of the UC patients were admitted for a flare, one for campylobacter-positive diarrhea and one for PR bleeding, resulting in early CS. The CD patient had two admissions for flares at 10/40 and 13/40.

Data on bio-chemical markers were available for a limited number of women during pregnancy. Mean CRP within two months prior to conception was 6.1 ± 9.6 mg/L (*n* = 59). The mean CRP during the T1, T2, T3 was 7.89 ± 17 mg/L (*n* = 49), 11.7 ± 21.1 mg/L (*n* = 48) and 11.4 ± 19.7 mg/L (*n* = 54), respectively. Mean FC within two months pre-conception was 255 ± 276.9 µg/g (*n* = 13). Mean FC in the T1, T2, T3 was 635 ± 754.6 µg/g, 442.6 ± 737.3 µg/g and 495.3 ± 528.1 µg/g, respectively.

### IBD Medications

Table 3 provides an overview of the IBD medications used in each trimester of pregnancy. Over ¾ of the cohort (102/130) were on IBD medication during pregnancy. Seventy-eight women (60%) were on 5-ASA therapy at conception, falling to 73 (56.2%) in T1, based on physician discretion. Twenty-five women were on a thiopurine at conception (azathioprine *n* = 23, 6-mercaptopurine *n* = 2) and a majority (18/25) continued a thiopurine throughout pregnancy. Eight patients were taking systemic corticosteroids at conception, while 26 were prescribed ≥ 1 course of corticosteroids (prednisolone *n* = 23, budesonide *n* = 3) during pregnancy.

**Table 3:** Inflammatory bowel disease therapies during pregnancy

IBD Therapy, by trimester	CD	UC	IBD-U	Total
Pregnancies, *n* (%)	54	75	1	130
IBD therapy during pregnancy, *n* (%) *Includes 5-ASA, thiopurine, biologic and systemic corticosteroids*	41 (75.9)	65 (86.7)	1 (100)	107 (82.3)
**5-ASA therapy**				
Conception, *n* (%)	21 (38.9)	56 (74.7)	1 (100)	78 (60)
T1, *n* (%)	20 (37.0)	52 (69.3)	1 (100)	73 (56.2)
T2, *n* (%)	19 (35.2)	53 (70.7)	1 (100)	73 (56.2)
T3, *n* (%)	18 (33.3)	56 (74.7)	1 (100)	75 (57.7)
**Biologic therapy**				
Conception, *n* (%)	19 (35.2)	5 (6.7)	0	24 (18.5)
T1, *n* (%)	19 (35.2)	5 (6.7)	0	24 (18.5)
T2, *n* (%)	19 (35.2)	5 (6.7)	0	24 (18.5)
T3, *n* (%)	16 (29.6)	5 (6.7)	0	21 (16.2)
**Thiopurine**				
Conception, *n* (%)	14 (25.9)	11 (14.7)	0	25 (19.2)
T1, *n* (%)	11 (20.4)	10 (13.3)	0	21 (16.2)
T2, *n* (%)	9 (16.7)	9 (12)	0	18 (13.8)
T3, *n* (%)	9 (16.7%)	9 (12)	0	18 (13.8)
**Corticosteroids (systemic)**				
Conception, *n* (%)	5 (9.3)	3 (4)	0	8 (6.2)
T1, *n* (%)	6 (11.1)	6 (8)	0	12 (9.2)
T2, *n* (%)	5 (9.3)	6 (8)	0	11 (8.5)
T3, *n* (%)	4 (7.4)	7 (9.3)	0	11 (8.5)

There were 26 patients on no therapy at all at the time of conception (CD = 13; UC = 13). Of the patients with Crohns, seven were in remission at conception and five had mild disease activity. There was insufficient data to allow PGA of disease activity for one patient. Eleven of these patients requires no therapy in pregnancy; two received steroids. A further two patients with mild disease activity on 5-ASA at conception had their 5-ASA stopped at conception and required no IBD therapies in pregnancy, including steroids. Of the 13 patients not requiring any therapy in pregnancy, there was one preterm delivery at 36 weeks’ gestation due to active CD in the setting of past perianal disease, two instances of minor congenital anomalies (camptodactyly and talipes) and one incidence of both SGA and LBW in a single infant. There were four episodes of neonatal infection (three non-serious, one serious) and three women experiencing IBD complications within 30 days post partum.

Of the 13 patients with UC not on therapy at conception, PGA assessment, possible for 10 of these at conception, concluded four had mildly active disease, two moderate and four were in remission. Six patients completed pregnancy without IBD therapy and seven received 5-ASA only during pregnancy. An additional fourwomen elected to stop their 5-ASA after a positive pregnancy test. Of these 10 patients not on therapy throughout the remainder of pregnancy, none required steroids either. There was one pre-term delivery at 35 weeks by elective CS due to a complex surgical history and the wish to avoid sphincteric injury due to planned future IPAA surgery. There was one incidence of LBW and SGA in a term infant, no congenital anomalies, one instance of neonatal infection (non-serious) and two instances of maternal IBD complications within 30 days post partum.

### Biologic therapy

Twenty-four patients (18.4%) were on biologic therapy at conception; 13 (10%) on adalimumab, 10 (7.7%) on infliximab and one (0.8%) on ustekinumab. Five of these patients had UC (infliximab *n* = 2; adalimumab *n* = 3) and 19 had CD (infliximab *n* = 8; adalimumab *n* = 10; ustekinumab *n* = 1). One patient commenced infliximab therapy in T2. Eight patients were on a combined biologic and immunomodulator; one patient with UC on infliximab with azathioprine and seven patients with CD on anti-TNF therapy with either azathioprine (*n* = 6) or mercaptopurine (*n* = 1). All but one maintained combined therapy throughout pregnancy; this patient chose to stop their azathioprine after a positive pregnancy test. One patient on combined therapy received prednisolone in T2 and T3 for UC; there was otherwise no steroid exposure in these patients.

One patient on ustekinumab at conception was switched to adalimumab in T1 due to non-response. There was documentation of discussion between clinician and patients regarding the risks and benefits of continuing or stopping biologic therapy in T3 in 23/24 patients (95.8%). Seventeen patients discontinued biologic therapy during pregnancy; sixteen on the advice of the IBD team in T3 to limit placental drug transfer and one patient stopped therapy unilaterally in early pregnancy. Statistical analysis and comparison of patients who either continued or stopped anti-TNF therapy in T3 is described in Supplementary Table D.

Table 4 summarizes the data on last dose of biologic administered during pregnancy. There was inadequate data on the timing of the last biologic dose prior to delivery for four patients on adalimumab, possibly due to patient self-administration of drug. All patients continued biologic treatment throughout T1 and T2, except one patient on adalimumab discontinuing the drug early in pregnancy out of personal choice. Last dose of biologic therapy was given at mean gestational age of 28 weeks and biologics were restarted post partum at median 57.5 days.

**Table 4:** Biologic cessation and resumption in pregnancy

Biologic dose timing in Pregnancy	Mean ± SD
Gestation of last dose biologic (weeks) – All	28.4 ± 7.0
Gestation of last dose biologic (weeks) – Infliximab	29.7 ± 2.7 (Range 26–34 weeks)
Gestation of last dose biologic (weeks) – Adalimumab	30.2 ± 1.1 (Range 2–32 weeks)
Mean days post partum of biologic restart	166.7 ± 256.7 (Range 4–944, median 57.5)

## Obstetric Considerations

T3 growth scans were carried out in 88/128 pregnancies (68.8%; two pregnancies excluded due to T2 miscarriage). Of the 44 women who were known to have remained in remission from conception to delivery, 26 (59.1%) had additional T3 growth scans. The indications for these were not available. Eighteen of the 28 women (64.3%) with a maximum of mild disease activity at any point during pregnancy received additional growth scans and 19 of the 21 women (90.5%) with at maximum of moderate disease activity at any point during pregnancy received additional growth scans in T3. All five women who experienced severe disease at any point during pregnancy had T3 growth scans.

Only 10 of the 26 women with either a maximum of moderate or severe disease activity during their pregnancy (38.5%) received VTE prophylaxis with low molecular weight heparin (LMWH).

### Delivery Mode

Table 5 summarizes the mode of delivery and overall perineal outcomes in our cohort. Overall, 91 women (71.0%) had a vaginal birth and 37 (28.9%) had CS (elective *n* = 20, emergency *n* = 17). There were 48 cases of perineal injuries overall and 16 cases of episiotomy. All emergency CS were performed for obstetric indications. These included pre-eclampsia (*n* = 2), eclampsia (*n* = 1), breech presentation (*n* = 1), abnormal fetal monitoring (*n* = 8), poor progress of labor (*n* = 1), oligohydramnios and SGA (*n* = 1), failed instrumental delivery (*n* = 2) and onset of labor prior to a planned elective CS (*n* = 1). Emergency CS was not associated with any individual IBD therapy (5-ASA *p* = 0.6849; thiopurines *p* = 0.2474, biologic *p* = 0.4629; corticosteroids *p* = 0.8321). There was also no statistically significant association between disease activity at conception or in any trimester and emergency CS (conception *p* = 0.6612; T1 *p* = 0.7736, T2 *p* = 0.9233, T3 *p* = 0.7559).

**Table 5:** Data on delivery mode and perineal outcomes

Mode of delivery in pregnancies continuing beyond 24 weeks	*n* = 128 (%)
Vaginal unassisted	71 (55.5)
Vaginal assisted	20 (15.6)
Elective Cesarean section	20 (15.6)
Emergency Cesarean section	17 (13.3)
**Perineal outcomes**	
No injury	66 (51.6) (all births) 28 (30.8) (all vaginal births assisted/unassisted)
Other	1 (peri-urethral tear)
First degree	14 (49.1)
Second degree	30 (23.4)
Third degree	2 (1.6)
Fourth degree	1 (0.8)
Episiotomy	16 (12.5)

Of the six patients in our cohort with a previous history of perianal CD, three underwent elective CS and three women had a vaginal delivery. The elective CS indications included active CD (*n* = 2) and a complex prior surgical history (*n* = 2). There was one patient in our cohort with active perianal CD and she had an elective CS for this reason. All five patients in our cohort with an ileostomy underwent elective CS. Two of these women experienced intra-operative complications (small bowel obstruction secondary to band adhesions requiring laparotomy and bladder damage requiring intra-operative repair).

Documented antenatal advice on mode of delivery from the IBD team was present in 20 cases (15.6%). In nine pregnancies there was specific and more complex documented discussion between obstetricians and the IBD team regarding mode of delivery. Of these nine cases, seven had a diagnosis of CD (two with previous history of perianal CD), four had a history of previous IBD-related bowel resection and two had an ileostomy. Six of these patients went on to have a CS, including both patients with a history of perianal CD and both patients with an ileostomy.

## Pregnancy outcomes

Overall, the pregnancy maternal and neonatal outcomes for our cohort are listed in Table 6. Mean gestational age at birth was 38.6 weeks; 11 pregnancies (8.6%) resulted in preterm delivery and there were three miscarriages (2.3%). The mean birth weight was 3188 g (range 1132-4480 g); 14 (10.9%) neonates were LBW. LBW was not associated with 5-ASA (*p* = 0.1873), thiopurine (*n* = 0.1829) or biologic therapy (*p* = 0.3046). Corticosteroid use in all trimesters was significantly associated with LBW (T1 *p* = 0.0164; T2 *p* = 0.0047; T3 *p* = 0.0075). SGA was present in eight infants (6.3%) and there was no association between SGA and 5-ASA (0.3930), corticosteroids (*p* = 0.7796) or biologic therapy (*p* = 0.5788). Thiopurine therapy during pregnancy was associated with SGA (*p* = 0.0173) but not with any individual trimester (T1 *p* = 0.4328; T2 *p* = 0.4975; T3 *p* = 0.4975). There were 13 instances of congenital anomalies spread between 11 infants (8.6%). These included Silver-Russell syndrome, trisomy 21 with atrioventricular septal defect, ventriculomegaly, heart murmur, scaphocephaly, bilateral clinodactyly, camptodactyly, talipes and tongue tie.

**Table 6:** Pregnancy maternal and neonatal outcomes

Outcome	*n* = 128 (%)
Miscarriage (< 24 weeks gestation)	3 (2.3)^1^
Pregnancy duration (weeks) (*n* = 128)^2^	Mean 38.6 ± SD 2.0
Pre-term delivery	11 (8.6)
Sex of neonate	Female 64 (50)
Congenital anomalies	11 (8.6)
Birth weight (gram)	Mean 3188.3 (SD 587.5)
Low birth weight (< 2500 g)	14 (10.9)
Small for gestational age	8 (6.3)

^1^ Two complete miscarriages plus one demise of twin at 8/40; remaining twin born live

^2^ Excluded complete miscarriages (*n* = 2), at 20/40 and 17/40

### Risk factors for adverse outcomes

We assessed the following adverse pregnancy and neonatal outcomes: preterm birth, emergency CS, LBW and congenital anomalies and assessed for any association with either disease activity or IBD therapy use during pregnancy.

Active IBD at conception (*p* = 0.04), during T1 (*p* = 0.03), T2 (*p* < 0.0001) and T3 (*p* = 0.0001) was significantly associated with preterm birth. Maternal steroid use during T2 (*p* = 0.02), but not during T1 and T3, was also associated with preterm birth. No other IBD therapy was associated with preterm birth (5-ASA *p* = 0.4754; thiopurines *p* = 0.1026, biologics *p* = 0.4367). Similarly, there was a trend towards active disease in T2 (*p* = 0.07) and T3 (*p* = 0.06) and LBW. Steroid use throughout gestation was also significantly associated with LBW (T1 *p* = 0.02, T2 *p* = 0.005, T3 *p* = 0.007). Neither 5-ASA (*p* = 0.4329), thiopurine (*p* = 0.6507) nor biologic therapy (0.6900) were associated with congenital anomaly.

### Post-partum

Of the 24 neonates exposed to biologic therapy in utero, there was documented evidence in 21 cases (87.5%) advising the mother regarding the importance of avoiding live vaccinations for at least the first six months of baby’s life. There were 27 cases of neonatal infections by six months, 11 serious (defined as requiring hospitalization) and 16 non-serious. This was not associated with in-utero 5-ASA (*p* = 0.0803), biologic (*p* = 0.7277) or thiopurine exposure (*p* = 0.5224). The only factors found to be significantly associated with neonatal infections were active disease (*p* = 0.0075) and steroid use (*p* = 0.0482) in T3.

Within 30 days post partum, 11 women (8.5%) experienced an IBD flare. A third of patients (31.5%) were reviewed by their gastroenterology team within two months post partum and 38.5% between two and six months post partum.

## Discussion

In this large tertiary experience of the management of pregnancies in women with IBD we noted a low incidence of adverse materno-fetal outcomes. The rates of pregnancy adverse outcomes are comparable to those reported in the large prospective observational study of 1490 IBD pregnancies (PIANO registry): 8.6% vs. 9% congenital anomalies, 2.3% vs. 3% miscarriage, 10.9% vs. 7% LBW and 8.6% vs. 10% pre-term birth [12].

International guidelines [5, 6, 9, 13] emphasize the importance of achieving remission prior to conception and continuation of suitable IBD medications to maintain remission throughout gestation. Over half of our cohort were in remission at conception and elevated disease activity was less frequent in these patients. We noted higher rates of active UC than CD during pregnancy as reported by Rottenstreich et al. (48% vs. 32%; *p* = 0.005) [14] and the EpiCom observational study [15]. Possible reasons for this could be that women with UC are being undertreated during pregnancy [16].

Patients with mild or moderate IBD activity at conception were more likely to experience active disease during pregnancy, higher rates of pre-term birth and LBW. A large French cohort study of 36,654 IBD pregnancies and the PIANO registry also reported that active IBD before and during pregnancy was associated with increased rates of prematurity, SGA and more stillbirths compared to non-IBD pregnancies [12, 17].

Most of our patients continued their IBD therapy during pregnancy in line with international recommendations [5, 6, 9, 13]. Anti-TNF therapy was continued in the vast majority of women through to T3, with most receiving their last dose towards the start of T3. There was no association with the use of 5-ASA, thiopurine or biologics during pregnancy and rates of pre-term birth, emergency CS, LBW, congenital anomalies or neonatal infections in the first six months of life.

BSG guidance advises that all pregnant women receiving biologic therapy should receive individualized advice on whether to stop or continue therapy in T3 [6]. We were compliant with documentation available in 95.8% cases. As anti-TNFs can be detected in the infant up to six to nine months after birth, caution is mandated with live vaccines and live vaccinations should be deferred until at least six to 12 months of age [6]. This was documented in 87.5% cases in our cohort and no infant received live vaccination prior to nine months after birth.

Corticosteroid exposure was associated with a greater risk of LBW in all trimesters and increased rates of serious neonatal infections in the first six months of life, pre-term birth in T2 exposure and neonatal infections in T3 exposure, similar to the PIANO registry (preterm birth [OR 1.5; 95% CI 1.0–2.3] and LBW [OR 1.8; 95% CI 1.1- 2.9]) [10].

Additional growth scans are advised in T3 in women with active IBD, as active disease increases the risk of LBW and SGA. These were performed accordingly in 64.3% of women with mild disease, 90.5% with moderate disease and 100% with severe disease activity [6]. Guidelines advise pregnant IBD patients are risk assessed for VTE and LMWH considered in those with active disease, hospitalized or following CS [5, 6]. In our study, only 38.5% of women with moderate or severely active disease during pregnancy were prescribed LMWH, emphasizing need for wider dissemination of this best practice.

Just under a third of patients (37/128; 28.9%) in our cohort delivered via CS. The rates of vaginal delivery, elective CS and emergency CS were comparable to national rates reported in England and Wales [18]. This is in contrast with a recent systematic review that CS was more common in patients with IBD compared to those without IBD (OR 1.79, 95% CI, 1.2–2.8) and the PIANO registry where a 44% CS rate was reported [10, 12, 19]. Similarly, perineal injury rates were comparable to national averages and episiotomy rates were lower than expected [18, 20].

We acknowledge some limitations of our study. Our patient numbers were lower than multi-centre prospective registries. As an inherent limitation of our retrospective study design, there was  some missing data and inconsistencies with the availability of biomarkers for many patients, limiting our objective assessment of disease activity and informing our choice to use physician global assessment for this purpose. Outcomes such as miscarriage rates are difficult to study as these can occur before the pregnancy is documented on secondary care medical records. We did not have patients on non-anti-TNF biologics in our cohort, yet 16% of our patients were anti-TNF treated which should provide added confidence to gastroenterologists to use these effective therapies in pregnancy when needed.

Despite our limitations, our real-world tertiary experience demonstrated consistency and uniformity in IBD and obstetric care. We assessed each pregnancy from the pre-conception through to the post-partum period and until neonate reached six months of age; in contrast to other studies where the post-partum period was not included [4, 21].

In conclusion, our findings demonstrate that the maternal and neonatal outcomes in pregnancies occurring in women with IBD in remission at conception are favorable and comparable to the general population. Active disease at conception and during pregnancy is associated with pre-term birth and LBW. IBD therapies, including anti-TNF and thiopurines, may be continued to ensure maintenance of remission. Corticosteroid treatment is associated with pre-term birth, LBW and neonatal infections. Optimal control of IBD pre-conception whenever possible and effective use of treatments supported by international guidelines is key for optimal outcomes for mother and baby.

## Supplementary Information

Below is the link to the electronic supplementary material.

Supplementary file1 (DOCX 19 KB)

Supplementary file2 (DOCX 18 KB)

Supplementary file3 (DOCX 19 KB)

Supplementary file4 (DOCX 20 KB)

## Data Availability

Data available upon reasonable request from the corresponding author [JKL].

## Declarations

### Disclaimer

The authors are solely responsible for the data and the contents of the paper. In no way, are the Editor-in-Chief, Editorial Board Members, the Indian Society of Gastroenterology or the printer/publishers responsible for the results/findings and content of this article.

## References

[1] Wang R, Li Z, Liu S, et al. Global, regional and national burden of inflammatory bowel disease in 204 countries and territories from 1990 to 2019: a systematic analysis based on the Global Burden of Disease Study 2019. BMJ Open. 2023;13:e065186. 10.1136/bmjopen-2022-065186. 10.1136/bmjopen-2022-065186 PMC10069527 36977543

[2] Molodecky NA, Soon IS, Rabi DM, et al. Increasing incidence and prevalence of the inflammatory bowel diseases with time, based on systematic review. Gastroenterology. 2012;142:46-54.e42. 10.1053/j.gastro.2011.10.001. 10.1053/j.gastro.2011.10.001 22001864

[3] Kim MA, Kim YH, Chun J, et al. The influence of disease activity on pregnancy outcomes in women with inflammatory bowel disease: A systematic review and meta-analysis. J Crohns Colitis. 2021;15:719–32. 10.1093/ecco-jcc/jjaa225. 10.1093/ecco-jcc/jjaa225 33175122

[4] Avni Biron I, Hayat L, Ollech JE, et al. Pregnancy outcomes in a cohort of patients with inflammatory bowel disease: Data from a multidisciplinary clinic in a tertiary center. J Clin Med. 2023;12:4120. 10.3390/jcm12124120. 10.3390/jcm12124120 PMC10299482 37373814

[5] Torres J, Chaparro M, Julsgaard M, et al. European Crohn’s and colitis guidelines on sexuality, fertility, pregnancy, and lactation. J Crohns Colitis. 2023;17:1–27. 10.1093/ecco-jcc/jjac115. 10.1093/ecco-jcc/jjac115 36005814

[6] Selinger C, Carey N, Cassere S, et al. Standards for the provision of antenatal care for patients with inflammatory bowel disease: guidance endorsed by the British Society of Gastroenterology and the British Maternal and Fetal Medicine Society. Frontline Gastroenterol. 2020;12:182–7. 10.1136/flgastro-2020-101459. 10.1136/flgastro-2020-101459 PMC8040496 33912332

[7] Liu E, Chatten K, Limdi JK. Conception, pregnancy and inflammatory bowel disease – Current concepts for the practising clinician. Indian J Gastroenterol. 2024. 10.1007/s12664-024-01563-9. 10.1007/s12664-024-01563-9 PMC13009062 38748381

[8] Wolloff S, Moore E, Glanville T, et al. Provision of care for pregnant women with IBD in the UK: the current landscape. Frontline Gastroenterol. 2020;12:487–92. 10.1136/flgastro-2020-101546. 10.1136/flgastro-2020-101546 PMC8515275 34712466

[9] Mahadevan U, Robinson C, Bernasko N, et al. Inflammatory bowel disease in pregnancy clinical care pathway: a report from the American gastroenterological association IBD parenthood project working group. Gastroenterology. 2019;156:1508–24. 10.1053/j.gastro.2018.12.022. 10.1053/j.gastro.2018.12.022 30658060

[10] Odufalu FD, Long M, Lin K, et al. PIANO Investigators from the Crohn’s and Colitis Foundation (CCF) Clinical Research Alliance recruited patients for their respective centers for participant enrollment. Exposure to corticosteroids in pregnancy is associated with adverse perinatal outcomes among infants of mothers with inflammatory bowel disease: results from the PIANO registry. Gut. 2022;71:1766–72. 10.1136/gutjnl-2021-325317. 10.1136/gutjnl-2021-325317 34686575

[11] Chugh R, Long MD, Jiang Y, Weaver KN, Beaulieu DB, Scherl EJ, Mahadevan U. Maternal and neonatal outcomes in vedolizumab and ustekinumab exposed pregnancies: results from the PIANO registry. Am J Gastroenterol. 2024;119:468–76. 10.14309/ajg.0000000000002553. 10.14309/ajg.0000000000002553 37796648

[12] Mahadevan U, Long MD, Kane SV, et al. Crohn’s colitis foundation clinical research alliance. pregnancy and neonatal outcomes after fetal exposure to biologics and thiopurines among women with inflammatory bowel disease. Gastroenterology. 2021;160:1131–9. 10.1053/j.gastro.2020.11.038. 10.1053/j.gastro.2020.11.038 PMC7956164 33227283

[13] Nguyen GC, Seow CH, Maxwell C, et al. IBD in pregnancy consensus group; canadian association of gastroenterology. The toronto consensus statements for the management of inflammatory bowel disease in pregnancy. Gastroenterology. 2016;150:734-57.e1. 10.1053/j.gastro.2015.12.003. 10.1053/j.gastro.2015.12.003 26688268

[14] Rottenstreich A, Fridman Lev S, Rotem R, et al. Disease flare at prior pregnancy and disease activity at conception are important determinants of disease relapse at subsequent pregnancy in women with inflammatory bowel diseases. Arch Gynecol Obstet. 2020;301:1449–54. 10.1007/s00404-020-05557-8. 10.1007/s00404-020-05557-8 32377786

[15] Pedersen N, Bortoli A, Duricova D, et al. European crohn-colitis organisation-ECCO-study group of epidemiology committee-EpiCom the course of inflammatory bowel disease during pregnancy and postpartum: a prospective European ECCO-EpiCom. Study of 209 pregnant women. Aliment Pharmacol Ther. 2013;38:501–12. 10.1111/apt.12412. 10.1111/apt.12412 23855425

[16] Nasef NA, Ferguson LR. Inflammatory bowel disease and pregnancy: overlapping pathways. Transl Res. 2012;160:65–83. 10.1016/j.trsl.2011.12.002. 10.1016/j.trsl.2011.12.002 22687963

[17] Meyer A, Drouin J, Weill A, et al. Pregnancy in women with inflammatory bowel disease: a French nationwide study 2010–2018. Aliment Pharmacol Ther. 2020;52:1480–90. 10.1111/apt.16074. 10.1111/apt.16074 33095502

[18] NMPA Project Team. National Maternity and Perinatal Audit: Clinical Report. Based on births in NHS maternity services in England and Wales between 1 April 2018 and 31 March 2019. London: RCOG; 2022. p. 2022.

[19] Tandon P, Govardhanam V, Leung K, et al. Systematic review with meta-analysis: risk of adverse pregnancy-related outcomes in inflammatory bowel disease. Aliment Pharmacol Ther. 2020;51:320–33. 10.1111/apt.15587. 10.1111/apt.15587 31912546

[20] Thiagamoorthy G, Johnson A, Thakar R, et al. National survey of perineal trauma and its subsequent management in the United Kingdom. Int Urogynaecol J. 2014;25:1621–7. 10.1007/s00192-014-2406-x. 10.1007/s00192-014-2406-x 24832856

[21] Innocenti T, Roselli J, Taylor A, et al. Pregnancy outcomes in inflammatory bowel disease: Data from a large cohort survey. J Dig Dis. 2022;23:473–81. 10.1111/1751-2980.13128. 10.1111/1751-2980.13128 PMC10092249 36156857

