# Supplementary material for: Pregnancy outcomes among women with inflammatory bowel disease: A UK tertiary centre experience
Source: Indian J Gastroenterol. 2024 Sep 2;45(2):230–9. doi: 10.1007/s12664-024-01657-4 (PMC13009036; doi:10.1007/s12664-024-01657-4)
Supplement: Supplementary file 1 — Supplementary file1 (DOCX 19 KB) [file 12664_2024_1657_MOESM1_ESM.docx]

| **Characteristic** | **E1 (Proctitis)**,  n = 22*^1^* | **E2 (Left-sided)**,  n = 25*^1^* | **E3 (Extensive)**,  n = 27*^1^* | **p-value***^2^* |
| --- | --- | --- | --- | --- |
| **Preterm Birth** | 2 (9.1%) | 3 (12%) | 2 (7.4%) | 0.9 |
| **C-Section (CS)** |  |  |  | 0.2 |
| Elective CS | 6 (75%) | 2 (25%) | 2 (50%) |  |
| Emergency CS | 2 (25%) | 6 (75%) | 2 (50%) |  |
| **Low Birth Weight** | 3 (14%) | 4 (16%) | 2 (7.4%) | 0.7 |
| **Congenital Anomalies** | 3 (14%) | 0 (0%) | 4 (15%) | 0.12 |
| **Small for Gestational Age** | 2 (9.1%) | 2 (8.0%) | 0 (0%) | 0.3 |
| **Neonatal Infections** |  |  |  | 0.8 |
| Non-serious | 2 (50%) | 1 (50%) | 5 (71%) |  |
| Serious | 2 (50%) | 1 (50%) | 2 (29%) |  |
| *^1^*n (%)  *^2^*Fisher's exact test | | | | |

**Supplementary Table A. UC disease extent and pregnancy outcomes**
