# Supplementary material for: Pregnancy outcomes among women with inflammatory bowel disease: A UK tertiary centre experience
Source: Indian J Gastroenterol. 2024 Sep 2;45(2):230–9. doi: 10.1007/s12664-024-01657-4 (PMC13009036; doi:10.1007/s12664-024-01657-4)
Supplement: Supplementary file 2 — Supplementary file2 (DOCX 18 KB) [file 12664_2024_1657_MOESM2_ESM.docx]

| **Characteristic** | **Ileal (L1)**,  n = 9*^1^* | **Colonic (L2)**,  n = 16*^1^* | **Ileocolonic (L3)**,  n = 29*^1^* | **p-value***^2^* |
| --- | --- | --- | --- | --- |
| **Preterm Birth** | 0 (0%) | 1 (6.3%) | 3 (11%) | 0.8 |
| **C Section (CS)** |  |  |  | 0.3 |
| Elective CS | 0 (0%) | 3 (60%) | 7 (70%) |  |
| Emergency CS | 2 (100%) | 2 (40%) | 3 (30%) |  |
| **Low Birth Weight** | 0 (0%) | 2 (13%) | 2 (7.1%) | 0.6 |
| **Congenital Anomalies** | 1 (11%) | 0 (0%) | 3 (11%) | 0.4 |
| **Small for Gestational Age** | 0 (0%) | 2 (13%) | 1 (3.6%) | 0.3 |
| **Neonatal Infections** |  |  |  | 0.5 |
| Non-serious | 1 (33%) | 2 (50%) | 5 (71%) |  |
| Serious | 2 (67%) | 2 (50%) | 2 (29%) |  |
| ^1^ n(%)  ^2^ Fisher’s exact test |  |  |  |  |

**Supplementary Table B. CD disease location and pregnancy outcome**
