# Supplementary material for: Pregnancy outcomes among women with inflammatory bowel disease: A UK tertiary centre experience
Source: Indian J Gastroenterol. 2024 Sep 2;45(2):230–9. doi: 10.1007/s12664-024-01657-4 (PMC13009036; doi:10.1007/s12664-024-01657-4)
Supplement: Supplementary file 3 — Supplementary file3 (DOCX 19 KB) [file 12664_2024_1657_MOESM3_ESM.docx]

| **Characteristic** | **Non-stricturing, non-penetrating (B1)**,  n = 37*^1^* | **Stricturing (B2)**,  n = 11*^1^* | **Penetrating (B3)**,  n = 6*^1^* | **p-value***^2^* |
| --- | --- | --- | --- | --- |
| **Preterm Birth** | 3 (8.1%) | 1 (10%) | 0 (0%) | >0.9 |
| **C-Section (CS)** |  |  |  | 0.4 |
| **Elective CS** | 6 (46%) | 2 (100%) | 2 (100%) |  |
| **Emergency CS** | 7 (54%) | 0 (0%) | 0 (0%) |  |
| **Low Birth Weight** | 4 (11%) | 0 (0%) | 0 (0%) | 0.7 |
| **Congenital Anomalies** | 1 (2.7%) | 1 (10%) | 2 (33%) | 0.043 |
| **Small for Gestational Age** | 3 (8.3%) | 0 (0%) | 0 (0%) | >0.9 |
| **Neonatal Infections** |  |  |  | 0.6 |
| **Non-serious** | 5 (56%) | 1 (33%) | 2 (100%) |  |
| **Serious** | 4 (44%) | 2 (67%) | 0 (0%) |  |
| *^1^*n (%) | | | | |
| *^2^*Fisher's exact test | | | | |

**Supplementary Table C. CD disease behaviour and pregnancy outcomes.**
