# Supplementary material for: Pregnancy outcomes among women with inflammatory bowel disease: A UK tertiary centre experience
Source: Indian J Gastroenterol. 2024 Sep 2;45(2):230–9. doi: 10.1007/s12664-024-01657-4 (PMC13009036; doi:10.1007/s12664-024-01657-4)
Supplement: Supplementary file 4 — Supplementary file4 (DOCX 20 KB) [file 12664_2024_1657_MOESM4_ESM.docx]

| **Characteristic** | **Not stopped**, n = 7*^1^* | **Stopped**, n = 16*^1^* | **p-value***^2^* |
| --- | --- | --- | --- |
| **Diagnosis** |  |  | 0.6 |
| **CD** | 5 (71%) | 13 (81%) |  |
| **UC** | 2 (29%) | 3 (19%) |  |
| **Preterm Birth** |  |  |  |
| **No** | 7 (100%) | 16 (100%) |  |
| **C-Section (CS)** |  |  | >0.9 |
| **Elective CS** | 1 (100%) | 4 (80%) |  |
| **Emergency CS** | 0 (0%) | 1 (20%) |  |
| **Low Birth Weight** |  |  |  |
| **No** | 7 (100%) | 16 (100%) |  |
| **Congenital Anomalies** | 0 (0%) | 1 (6.3%) | >0.9 |
| **Small Gestational Age** |  |  |  |
| **No** | 7 (100%) | 16 (100%) |  |
| **Neonatal Infections** |  |  | >0.9 |
| **Non-serious** | 1 (50%) | 3 (60%) |  |
| **Serious** | 1 (50%) | 2 (40%) |  |
| **PGA Disease Activity T1** |  |  | 0.7 |
| **Remission** | 6 (86%) | 13 (87%) |  |
| **Mild** | 0 (0%) | 1 (6.7%) |  |
| **Moderate** | 1 (14%) | 0 (0%) |  |
| **Severe** | 0 (0%) | 1 (6.7%) |  |
| **PGA Disease Activity T2** |  |  | 0.2 |
| **Remission** | 6 (86%) | 13 (81%) |  |
| **Mild** | 0 (0%) | 3 (19%) |  |
| **Moderate** | 0 (0%) | 0 (0%) |  |
| **Severe** | 1 (14%) | 0 (0%) |  |
| **PGA Disease Activity T3** |  |  | 0.6 |
| **Remission** | 6 (86%) | 11 (69%) |  |
| **Mild** | 1 (14%) | 5 (31%) |  |
| **Moderate** | 0 (0%) | 0 (0%) |  |
| **Severe** | 0 (0%) | 0 (0%) |  |
| *^1^*n (%) | | | |
| *^2^*Fisher's exact test | | | |

**Supplementary Table D. Comparison of patients continuing vs. stopping biologic therapy in T3**
